# Sex differences in baseline characteristics, stroke care, and outcomes across socioeconomic settings: a multicentre study from Switzerland, Poland, and Ukraine

**DOI:** 10.1007/s00415-026-13616-4

**Published:** 2026-02-17

**Authors:** Charlotte Berger, Helly Hammer, Marino Costa, Pawel Lowiec, Andriy Yagensky, Adrian Scutelnic, Kateryna Antonenko, Olga Biletska, Bartosz Karaszewski, Hakan Sarikaya, Tomasz Zdrojewski, Anastasiia Klymiuk, Claudio LA. Bassetti, Natalia Yashchuk, Kamil Chwojnicki, Marcel Arnold, Hugo Saner, Mirjam R. Heldner

**Affiliations:** 1 Department of Neurology, Inselspital, University Hospital and University of Bern, Bern, Switzerland; 2 Department of Neurology, Medical University of Gdansk, Gdansk, Poland; 3 Regional Center for Cardiovascular Disease, Communal Enterprise “Medical Association of Lutsk City Territorial Community”, Lutsk, Ukraine; 4 Department of Cardiovascular Prevention, Medical University of Gdansk, Gdansk, Poland; 5 Department of Anaesthesiology and Intensive Care, Medical University of Gdansk, Gdansk, Poland; 6 Institute for Social and Preventive Medicine, University of Bern, Bern, Switzerland

**Keywords:** Sex differences, Stroke, Outcome, Secondary prevention, Socioeconomic environments

## Abstract

**Introduction:**

While stroke risk and burden vary across Europe, the combined impact of sex and socioeconomic context on stroke care and recovery remains poorly understood.

**Patients and methods:**

This observational multicentre cohort study analyzed prospectively collected registry data from patients with acute ischemic stroke from three tertiary care hospitals in different socioeconomic settings: Bern/Switzerland (high-income region; women = 128/293(43.7%)), Gdansk/Poland (high-income region; women = 54/139(38.8%)), and Lutsk/Ukraine (low-income region; women = 86/188(45.7%)).

**Results:**

We analyzed the data of 620 patients (median age CH-W/M:77.4/71.2;PL-W/M:75.5/67.0;UA-W/M:73.0/67.0;*p*-all = 0.051/*p*-W = 0.434/*p*-M = 0.130) admitted between 7 and 12/2019. There were significant differences between countries in women’s pre-stroke mRS: 0–1 (women: Switzerland: 73.6%/Poland: 62.9%; *p*-all/women < 0.001) and BMI (women: Switzerland: 24.2/Poland: 27.9/Ukraine: 29.3; *p*-women < 0.001). Stroke severity differed overall and between sexes (median NIHSS women: Switzerland: 4/Poland: 13/Ukraine: 7, men: Switzerland: 4/Poland: 9/Ukraine: 7; *p*-all < 0.001; *p*-women/men = 0.005). Atrial fibrillation was more common in women (women: Switzerland: 31.3%/Poland: 57.4%/Ukraine: 47.7%) vs. men (men: Switzerland: 26.1%/Poland: 31.8%/Ukraine: 32.4%; *p*-all = 0.006/*p*-women = 0.002), as was arterial hypertension (*p*-all/women/men < 0.001), with the highest rates in Ukraine (all: 87.2%). Smoking status differed significantly overall (*p*-all < 0.001), with the lowest rates in Ukrainian women (never smokers: 96.5%) and highest in Swiss men (active smokers: 40.7%).

Initial neuroimaging differed significantly by country, with the highest MRI use in Swiss women (women: Switzerland: 49.6%/Poland: 14.8%/Ukraine: 16.3%), highest CT use in Ukrainian men (men: Switzerland: 25.8%/Poland: 45.9%/Ukraine: 72.5%) and lowest use of both modalities in Ukraine (*p*-all/women/men < 0.001). Therapy (IVT/EVT/craniectomy/conservative) also differed significantly (*p*-all/women/men < 0.001), with conservative therapy most common in Ukraine (women: 96.5%/men: 94.1%).

3-month excellent outcome was significantly higher in Swiss vs. Ukrainian women (adjusted-OR-Swiss–Ukrainian-women = 2.96; adjusted-*p*-women = 0.015). 3-month mortality did not differ significantly. 3-month smoking rates were higher in Switzerland vs. Ukraine (adjusted-OR-Swiss–Ukrainian-women = 9; adjusted-*p*-Swiss–Ukrainian-women = 0.05; adjusted-OR-Swiss–Ukrainian-men = 9.75; adjusted-*p*-Swiss–Ukrainian-men = 0.004).

**Discussion and conclusion:**

Swiss versus Ukrainian women were three times more likely to achieve excellent 3-month outcomes, associated with disparities in prevention strategies. These findings highlight the need for sex-, gender-, and socioeconomic-specific approaches to stroke prevention, with tailored interventions to reduce inequalities.

## Introduction

### Sex differences in stroke

Stroke is the third leading cause of death and disability worldwide, and its burden continues to rise, particularly in aging populations [1]. Understanding sex differences in stroke risk, treatment, and outcomes is essential to improving equity in care. These differences are shaped not only by biological and hormonal factors, but also by disparities in healthcare access, cultural norms and socioeconomic context.

Women have a higher lifetime risk of stroke, experience more severe strokes, and have worse post-stroke outcomes compared to men. Atrial fibrillation (AF), more prevalent in older women, contributes substantially to this increased risk [2, 3]. Moreover, sex-specific factors—such as hormonal influences, pregnancy-related conditions, and differences in symptom presentation—can complicate timely recognition and treatment [4–7].

While the biological aspects of sex differences are well documented, the influence of socioeconomic and regional healthcare disparities on these differences is less understood. Women may face additional barriers in accessing acute stroke therapy due to factors such as older age at presentation, greater pre-stroke disability, social isolation, and biases in healthcare delivery [8–13].

Differences in stroke care and outcomes are particularly pronounced across Europe. In 2019, ischaemic stroke accounted for 62.5% of all incident strokes globally, with country-specific variations (CH 66.95%, PL: 74.29%, and UA: 68.71%) [1]. High-income countries like Switzerland typically have well-established stroke units, high availability of advanced imaging and reperfusion therapy, and robust secondary prevention programs. In contrast, countries in Eastern Europe, including Ukraine, often face infrastructural challenges such as limited access to MRI or EVT, delayed treatment initiation, and fewer resources for rehabilitation and follow-up care [14–16].

Poland, a high-income EU member state with a strong public health system, represents an intermediate setting, with more standardized stroke care than Ukraine, but still differing from the Swiss model in terms of resources, guideline adherence, and care continuity [17–19].

Importantly, sex differences may manifest differently across these healthcare systems. For example, in settings where access is limited, older women with greater pre-stroke disability may be disproportionately excluded from advanced treatments. Similarly, prevention strategies such as atrial fibrillation screening, smoking cessation programs, or secondary prevention adherence vary substantially by country and may influence sex-specific outcomes [20–23].

To address these disparities, the European Stroke Action Plan 2018–2030 aims to standardize stroke care across socioeconomic contexts [24].

### Aim of this manuscript

Against this background, the present study aimed to investigate sex differences in baseline characteristics, acute stroke treatment, outcomes, and secondary prevention across three countries with distinct socioeconomic and healthcare environments: Switzerland (CH), Poland (PL), and Ukraine (UA). The goal was to explore how sex and socioeconomic context interact to influence stroke care and to identify modifiable factors that may inform more equitable, context-specific strategies across Europe.

## Methods

This is an observational, multicentre cohort analysis of patients with a first acute ischaemic stroke (AIS) (no other stroke types were analyzed) treated between July and December 2019 in three tertiary care centres. These centres are comparable in service population and research engagement, but might be above the national average in terms of quality. Data were collected consecutively in Bern/CH, Gdansk/PL and Lutsk/UA, all from three prospective stroke registries. Information was collected at baseline, discharge, and at 3-month follow-up. The identification of large vessel occlusions (LVOs) was based on CTA, MRA, and/or DSA imaging performed at admission, and the affected vessels were recorded. LVOs were defined according to current international guidelines as follows: internal carotid artery (ICA); the first and second segments of the middle cerebral artery (MCA: M1, M2); basilar artery (BA); vertebral artery (VA); the first and second segments of the posterior cerebral artery (PCA: P1, P2); and the first segment of the anterior cerebral artery (ACA: A1) [25]. Further details are provided in Berger C et al. 2024 [26]. The data set for this analysis was dichotomized by sex.

### Statistical analysis

Statistical analyses were conducted using SPSS version 25.0 (SPSS Inc., Chicago, Illinois, USA). The *χ*^2^ test was applied for categorical variables, while ANOVA was used for ordinal and continuous variables to evaluate baseline characteristics and outcomes between male and female patients from Switzerland, Poland, and Ukraine. A two-tailed *p*-value of < 0.05 was regarded as statistically significant. Outcome analysis employed binary logistic regression, along with ordinal and linear regression models, as appropriate. These regression analyses were adjusted for variations in admission National Institutes of Health Stroke Scale (NIHSS) scores and treatment approaches (conservative and/or mechanical thrombectomy and/or rtPA and/or craniectomy), as these factors differed significantly at baseline comparisons [27]. Age did not differ significantly between cohorts and pre-stroke morbidity data were partially unavailable, and thus were therefore not included as adjustment variables.

### Standard protocol approvals, registrations, patient consent, and reporting

The Bernese Stroke Registry, approved by the local ethics committee (KEK Bern 2016–01905), adheres to Swiss law, excluding patients who declined data usage for research, as previously described. Ethics approvals and written informed consent were obtained for participants in Poland and Ukraine. The study complies with the Declaration of Helsinki and follows STROBE guidelines for data analysis [28]. Further details are provided in Berger et al. 2024 [26]. Anonymized data not published within this article will be made available upon request from any qualified investigator.

## Results

### Baseline characteristics and reperfusion treatment

Data from 293 patients (43.7%: female/56.3%: male) in CH, 139 patients (38.8%: female/61.2%: male) in PL, and 188 patients (45.7%: female/54.3%: male) in UA were collected. Age, diabetes mellitus, wake up stroke, and TOAST (Trial of ORG 10172 in Acute Stroke Treatment [29]) aetiology were similar. There were significant differences in pre-morbid mRS distributions across all groups and specifically among women (*p*-all/W < 0.001), but not among men (*p*-M = 0.068), with most patients in both sexes and countries having a pre-morbid mRS of 0 (Switzerland: W: 60.8%/M: 64.4%, Poland: W: 29.6%/M: 52.9%) (no data were collected in Ukraine). There were body mass index (BMI) differences overall and among women (*p*-all/W < 0.001), but not among men (*p*-M = 0.442), with the lowest median BMI (24.2) in Swiss women and the highest in Ukrainian women (29.3). AF showed significant differences overall (*p*-all = 0.006) and among women (*p*-W = 0.002), but not among men (*p*-M = 0.462). The highest rates were found in Polish women (57.4%), and lowest in Swiss men (26.1%). Arterial hypertension differed significantly overall (*p*-all < 0.001) and by sex (*p*-W/M < 0.001), with highest rates in Ukraine (W: 87.2%/M: 87.3%). Hyperlipidaemia differed significantly (*p*-all/W/M < 0.001), with the lowest rates in Ukraine (W: 14%/M: 19.6%). Previous myocardial infarction (MI) differed by sex (*p*-W = 0.041/*p*-M = 0.046), with the highest rates in Polish women (16.7%) and Ukrainian men (20.7%). Smoking status differed significantly (*p*-all/W/M < 0.001). In Ukraine, most women were never smokers (96.5%), while the highest proportion of men who were former smokers was also found in Ukraine (55.4%). The highest proportion of active smokers was among Swiss men (40.7%). Initial NIHSS scores differed significantly (*p*-all < 0.001, *p*-W/M = 0.005). Both sexes in Poland had the highest median NIHSS score (W: PL: 13/CH: 4/UA: 7 and M: PL: 9/CH: 4/UA: 7). Initial neuroimaging differed significantly (*p*-all/W/M < 0.001). Swiss (49.6%) vs. Polish/Ukrainian (14.8%/16.3%) women were examined with MRIs more frequently. Ukrainian (72.5%) vs. Swiss/Polish (25.8%/45.9%) men were examined with CT scans more frequently. Ukrainian patients least often were examined with both modalities.

LVOs were present at similar frequencies in women and men in CH and PL (W: CH: 59.4%, PL: 59.3%; M: CH: 54.5%, PL: 57.6%; *p*-all = 0.751, *p*-W = 0.988, *p*-M = 0.640). Data for UA were not available. In women, the most common LVO site was M1 (CH: 24.2%, PL: 31.5%), followed by M2 and ICA. Other sites (A1, BA, VA, P1, P2) were less frequent. The distribution did not differ significantly between countries, in women (*p*-W = 0.459) or in men (*p*-M = 0.134), but overall (*p* = 0.026).

Stroke aetiology differed significantly (*p*-all/W/M < 0.001), with the highest rates of large artery atherosclerosis observed in Ukraine (W: UA: 48.8%/CH: 15.0%/PL: 5.6%; M: UA: 64.7%/CH: 12.1%/PL: 23.5% ) and higher rates of cardioembolism in women. Therapy differed significantly (*p*-all/W/M < 0.001). Notably, conservative therapy was most common in Ukraine (W: 96.6%/M: 94.1%). Further details are provided in Table 1.

**Table 1:** Baseline characteristics and reperfusion treatment

	Women			Men			*p*-value (Pearson)
	CH *n* = 128	POL *n* = 54	UA *n* = 86	CH *n* = 165	POL *n* = 85	UA *n* = 102	
Age [y] [median(range)]	77.4 (28.1–97.4)	75.5 (18.0–84.0)	73.0 (51.0–92.0)	71.2 (20.0–91.8)	67.0 (36.0–85.0)	67.0 (49.0–92.0)	all = 0.051/women = 0.434/men = 0.130
Time of the day [*n* (%)]							all = 0.589/women = 0.335/men = 0.975
Day	103 (80.5%)	39 (72.2%)	63 (73.3%)	124 (75.2%)	63 (74.1%)	77 (75.5%)	
Wake up stroke	25 (19.5%)	15 (27.8%)	23 (26.7%)	41 (24.8%)	22 (25.9%)	25 (24.5%)	
Pre-mRS [median (range)]	0 (0–4)	1 (0–3)	NA	0 (0–5)	0 (0–4)	NA	all < 0.001/women < 0.001/men = 0.068
0	76 (60.8%)	16 (29.6%)	NA	103 (64.4%)	45 (52.9%)	NA	
1	16 (12.8%)	18 (33.3%)	NA	31 (19.4%)	24 (28.2%)	NA	
2	8 (6.4%)	13 (24.1%)	NA	9 (5.6%)	12 (14.1%)	NA	
3	18 (14.4%)	7 (13.0%)	NA	9 (5.7%)	2 (2.4%)	NA	
4	7 (5.6%)	0 (0%)	NA	7 (4.4%)	2 (2.4%)	NA	
5	0 (0%)	0 (0%)	NA	1 (0.6%)	0 (0%)	NA	
BMI [median(range)]	24.2 (16.1–46.9)	27.9 (20.3–42.5)	29.3 (18.5–49.5)	27.0 (15.9–45.6)	27.7 (19.5–41.5)	27.3 (18.0–45.0)	All < 0.001/women < 0.001/men = 0.442
Atrial fibrillation	40 (31.3%)	31 (57.4%)	41 (47.7%)	43 (26.1%)	27 (31.8%)	33 (32.4%)	All = 0.006/women = 0.002/men = 0.462
Diabetes mellitus	26 (20.3%)	14 (25.9%)	20 (19.6%)	40 (24.2%)	12 (14.1%)	20 (19.6%)	All = 0.594/women = 0.642/men = 0.165
Arterial hypertension	79 (61.7%)	41 (75.9%)	75 (87.2%)	108 (65.5%)	60 (70.6%)	89 (87.3%)	All < 0.001/women < 0.001/men < 0.001
Hyperlipidaemia	107 (83.6%)	42 (77.8%)	12 (14.0%)	14 (86.0%)	65 (76.5%)	20 (19.6%)	All < 0.001/women < 0.001/men < 0.001
Previous myocardial infarction	8 (6.3%)	9 (16.7%)	5 (5.8%)	16 (9.8%)	12 (14.1%)	21 (20.7%)	All = 0.055 all/women = 0.041/men = 0.046
Smoking status							All < 0.001/women < 0.001/men < 0.001
Never smoker	54 (55.1%)	30 (56.6%)	82 (96.5%)	47 (34.8%)	25 (29.4%)	24 (26.1%)	
Active smoker	20 (20.4%)	9 (17.0%)	1 (1.2%)	55 (40.7%)	29 (34.1%)	17 (18.5%)	
Former smoker	24 (24.5%)	14 (26.4%)	2 (2.4%)	33 (24.4%)	31 (36.5%)	51 (55.4%)	
NIHSS initial [median (range)]	4 (0–36)	13 (1–33)	7 (2–25)	4 (0–36)	9 (0–28)	7 (1–30)	All < 0.001/women = 0.005/men = 0.005
Initial neuroimaging							All < 0.001/women < 0.001/men < 0.001
MRI	62 (49.6%)	8 (14.8%)	14 (16.3%)	83 (52.2%)	14 (16.5%)	17 (16.7%)	
CT	33 (26.4%)	32 (59.3%)	60 (69.8%)	41 (25.8%)	39 (45.9%)	74 (72.5%)	
Both	30 (24.0%)	14 (25.9%)	12 (14.0%)	35 (37.6%)	32 (37.6%)	11 (10.8%)	
Large vessel occlusion	76 (59.4%)	32 (59.3%)	NA	90 (54.5%)	49 (57.6%)	NA	All = 0.751/women = 0.988/men = 0.640
Large vessel occlusion main occlusion site							All = 0.026/women = 0.459/men = 0.134
ICA	14 (10.9%)	8 (14.8%)	NA	18 (10.9%)	18 (21.2%)	NA	
M1	31 (24.2%)	17 (31.5%)	NA	24 (14.5%)	15 (17.6%)	NA	
M2	20 (15.6%)	4 (7.4%)	NA	33 (20.0%)	11 (12.9%)	NA	
A1	1 (0.8%)	0 (0%)	NA	0 (0%)	0 (0%)	NA	
BA	3 (2.3%)	3 (5.6%)	NA	4 (2.4%)	4 (4.7%)	NA	
VA	4 (3.1%)	0 (0%)	NA	4 (2.4%)	1 (1.2%)	NA	
P1	1 (0.8%)	0 (0%)	NA	4 (2.4%)	0 (0%)	NA	
P2	2 (1.6%)	0 (0%)	NA	3 (1.8%)	0 (0%)	NA	
None	52 (40.6%)	22 (40.7%)	NA	75 (45.5%)	36 (42.4%)	NA	
Aetiology TOAST						A	all < 0.001/women < 0.001/men < 0.001
Large artery atherosclerosis	19 (15.0%)	3 (5.6%)	42 (48.8%)	20 (12.1%)	20 (23.5%)	66 (64.7%)	
Cardioembolism	52 (40.9%)	31 (57.4%)	41 (47.7%)	55 (33.3%)	30 (35.3%)	30 (29.4%)	
Small vessel occlusion	5 (3.9%)	1 (1.9%)	0 (0%)	14 (8.5%)	1 (1.2%)	0 (0%)	
Stroke of other determined aetiology	10 (7.9%)	3 (5.6%)	0 (0%)	16 (9.7%)	7 (8.2%)	0 (0%)	
Stroke of undetermined aetiology (cryptogenic stroke)	41 (32.3%)	16 (29.6%)	3 (3.5%)	60 (36.4%)	27 (31.8%)	6 (5.9%)	
Aetiology cardioembolism							all = 0.339/women = 0.122/men = 0.673
Cardioembolism	23 (42.6%)	75 (59.1%)	45 (52.3%)	110 (66.7%)	55 (64.7%)	72 (70.6%)	
No cardioembolism	31 (57.4%)	52 (40.9%)	41 (47.7%)	55 (33.3%)	30 (35.3%)	30 (29.4%)	
Therapy							all < 0.001/women < 0.001/men < 0.001
Conservative	46 (35.9%)	16 (29.6%)	83 (96.6%)	54 (32.7%)	37 (43.5%)	96 (94.1%)	
Intravenous thrombolysis with rtPA only	26 (20.3%)	14 (25.9%)	2 (2.3%)	40 (24.2%)	10 (11.8%)	5 (4.9%)	
EVT only	38 (29.7%)	9 (16.7%)	0 (0%)	51 (30.9%)	13 (15.3%)	1 (1.0%)	
Both rtPA and EVT	18 (14.1%)	14 (25.9%)	1 (1.2%)	18 (10.9%)	24 (28.2%)	0 (0%)	
Craniectomy only	0 (0%)	1 (1.9%)	0 (0%)	2 (1.2%)	0 (0%)	0 (0%)	
IVT, EVT, and craniectomy	0 (0%)	0 (0%)	0 (0%)	0 (0%)	1 (1.2%)	0 (0%)	

Sex PL: *n* = 139, data of 1 patient missing, pre-mRS CH: *n* = 285, data of 8 patients missing, smoking status CH: *n* = 233, data of 60 patients missing, PL: *n* = 139, data of 1 patient missing, UA: *n* = 177, data of 11 patients missing, initial neuroimaging CH: *n* = 284, data of 9 patients missing

### Outcome and medication at discharge

Excellent outcome differed significantly (*p*-all/W/M-*p* < 0.001). Swiss (47.3%) vs. Polish/Ukrainian (25.9%/8.1%) women had higher rates, as well as Swiss (48.0%) vs. Polish/Ukrainian (38.8%/14.7%) men.

Favourable outcome differed significantly overall (*p*-all < 0.001), and by sex (*p*-W = 0.004/*p*-M = 0.002). For both sexes, Swiss (W: 52.7%; M: 65.1%) vs. Polish/Ukrainian (W: 42.6%/29.1%;M: 57.9%/43.1%) had higher rates. Death was similar. Discharge destination differed significantly (*p*-all/W/M-*p* = < 0.001). Ukrainian women were most likely to be discharged home (90.5%), Swiss women least (18%). Hospital stay duration differed significantly overall (*p*-all < 0.001) and by sex (*p*-W < 0.001/*p*-M = 0.011). Swiss (3 days) vs. Polish/Ukrainian (8/10 days) women had the shortest stays. Men followed a similar pattern (CH: 3.9/PL: 8/UA: 10 days). Use of antihypertensives differed significantly (*p*-all/W/M < 0.001). Their use was highest in Ukraine (W: 89.3%/M: 95.7%) and lowest in Switzerland (W: 64.3%/M: 66%). Use of oral anticoagulants (OACs) and antiplatelets was similar. Use of statins differed significantly overall (*p*-all = 0.002) and in men (*p*-M = 0.020). Their use was highest in Polish (W: 93.8%/M: 97.4%). Use of oral antidiabetics and insulin was similar.

After adjustment: For both sexes, Swiss/Polish had similar excellent outcomes. However, Swiss vs. Ukrainian women and men were more likely to have an excellent outcome (adjusted-OR-W = 8.32/adjusted-*p*-W < 0.001; adjusted-OR-M = 4.8/adjusted-*p*-M < 0.001). Favourable outcome and death were similar. Significant differences were observed in discharge destination across all countries for both sexes (adjusted-*p*-W/M < 0.001). Hospital stay duration was shorter for Swiss vs. Polish/Ukrainian women (adjusted-*p*-W/M < 0.001), with no significant differences for men.

For antihypertensives, Swiss vs. Polish/Ukrainian women were significantly less likely to use them (adjusted-OR-CH-PL-W = 0.35/adjusted-*p*-CH-PL-W = 0.020; adjusted-OR-CH-UA-W = 0.29/adjusted-*p*-CH-UA-W = 0.012). Swiss vs. Polish/Ukrainian men were also less likely to use antihypertensives (adjusted-OR-CH-PL-M = 0.46/adjusted-*p*-CH-PL-M = 0.024; adjusted-OR-CH-UA-M = 0.07/adjusted-*p*-CH-UA-M < 0.001). Use of OACs, antiplatelets, and insulin was similar. For oral antidiabetics, Swiss vs. Ukrainian women had a lower likelihood of use (adjusted-OR-W = 0.20/adjusted-*p*-W = 0.007), while no significant differences were observed for men. Swiss vs. Polish  use of statins was significantly lower for women (adjusted-OR-W = 0.25/adjusted-*p*-W = 0.038) and men (adjusted-OR-M = 0.13/adjusted-*p*-M = 0.008). Further details are provided in Tables 2 and 3 and Supplemental Fig. 1.

**Table 2:** Outcome and medication at discharge according to sex

	Women			Men			*p*-value (Pearson)
	CH	POL	UA	CH	POL	UA	
mRS discharge							
Excellent 0–1	53 (47.3%)	14 (25.9%)	7 (8.1%)	73 (48.0%)	33 (38.8%)	15 (14.7%)	All < 0.001/women < 0.001/men < 0.001
Favourable 0–2	59 (52.7%)	23 (42.6%)	25 (29.1%)	99 (65.1%)	49 (57.9%)	44 (43.1%)	All < 0.001/women = 0.004/men = 0.002
Dead 6	9 (8.0%)	6 (11.1%)	2 (2.3%)	15 (9.9%)	10 (11.8%)	10 (9.8%)	All = 0.263/women = 0.883/men = 0.881
Mortality in hospital	9 (7.0%)	6 (11.1%)	2 (2.3%)	15 (9.1%)	19 (11.8%)	10 (9.8%)	All = 0.251/women = 0.105/men = 0.798
Discharge destination							All < 0.001/women < 0.001/men < 0.001
Home	23 (18.0%)	27 (50.0%)	76 (90.5%)	43 (26.2%)	46 (54.1%)	72 (78.3%)	
Rehabilitation	26 (20.3%)	14 (25.9%)	8 (9.5%)	45 (27.4%)	24 (28.2%)	20 (21.7%)	
Nursing facility	4 (3.1%)	7 (13.0%)	0 (0%)	0 (0%)	5 (5.9%)	0 (0%)	
Other acute care hospital	66 (51.6%)	0 (0%)	0 (0%)	61 (37.2%)	0 (0%)	0 (0%)	
Dead	9 (7.0%)	6 (11.1%)	0 (0%)	15 (9.1%)	10 (11.8%)	0 (0%)	
Duration first hospital [days] [median (range)]	3.02 (0–21)	8 (3–98)	10 (3–21)	3.94 (0–59)	8 (48–98)	10 (1–32)	All < 0.001/women < 0.001/all < 0.011
Antihypertensives	74 (64.3%)	40 (83.3%)	75 (89.3%)	99 (66.0%)	61 (80.3%)	88 (95.7%)	All < 0.001/women < 0.001/all < 0.011
OACs	38 (32.2%)	22 (45.8%)	29 (34.5%)	45 (29.8%)	19 (25.0%)	27 (29.3%)	All = 0.907/women = 0.243/men = 0.736
Antiplatelets	77 (64.7%)	27 (56.3%)	54 (64.3%)	104 (69.3%)	61 (80.3%)	61 (66.3%)	All = 0.589/women = 0.564/men = 0.113
Oral antidiabetics	12 (10.1%)	6 (12.5%)	15 (17.9%)	19 (12.6%)	12 (15.8%)	14 (15.2%)	All = 0.310/women = 0.269/men = 0.756
Insulin	10 (8.4%)	8 (16.7%)	4 (4.8%)	15 (9.9%)	2 (2.6%)	4 (4.3%)	All = 0.178/women = 0.066/men = 0.066
Statins	95 (79.8%)	45 (93.8%)	70 (83.3%)	129 (85.4%)	74 (97.4%)	83 (90.2%)	All = 0.002/women = 0.088/men = 0.020

For 18/15/12 Swiss/Polish/Ukrainian patients, who died during in-hospital care, no medication was assessed

mRS discharge CH: *n* = 264, data of 39 patients missing, discharge destination CH: *n* = 292, data of 1 patient missing, Antihypertensives CH: *n* = 265, data of 10 patients missing, OACs/antiplatelets/statins/oral antidiabetics/insulin CH: *n* = 270, data of 5 patients missing

**Table 3:** Outcome and medication at discharge according to sex

	Women				Men			
	*p*-value (adjusted CH-PL)	Adjusted odds ratio, CH-PL 95% CI	*p*-value (adjusted CH-UA)	Adjusted odds ratio, CH-UA 95% CI	*p*-value (adjusted CH-PL)	Adjusted odds ratio, CH-PL 95% CI	*p*-value (adjusted CH-UA)	Adjusted odds ratio, CH-UA 95% CI
mRS-discharge								
Excellent 0–1	0.477	1.36 (0.58–3.17)	< 0.001	8.32 (2.93–23.64)	0.773	0.91 (0.46–1.77)	< 0.001	4.8 (2.13–10.82)
Favourable 0–2	0.503	0.76 (0.34–1.70)	0.354	1.5 (0.64–3.56)	0.321	0.7 (0.35–1.42)	0.178	1.78 (0.77–4.14)
Dead 6	0.988	1.01 (0.32–3.19)	0.692	1.54 (0.18–13.00)	0.703	1.2 (0.47–3.12)	0.565	0.6 (0.10–3.45)
Mortality in hospital	0.744	0.83 (0.26–2.59)	0.768	1.34 (0.17–11.47)	0.387	0.47 (0.09–2.60)	0.938	1.04 (0.41–2.65)
Discharge destination	< 0.001	NA	< 0.001	NA	< 0.001	NA	< 0.001	NA
Duration first hospital [days] [median (range)]	< 0.001	NA	< 0.001	NA	0.387	NA	0.707	NA
Antihypertensives	0.020	0.35 (0.14–0.85)	0.012	0.29 (0.11–0.77)	0.024	0.46 (0.24–0.90)	< 0.001	0.07 (0.02–0.23)
OACs	0.147	0.59 (0.29–1.21)	0.712	0.86 (0.39–1.89)	0.305	1.4 (0.74–2.68)	0.462	1.32 (0.63–2.75)
Antiplatelets	0.465	1.3 (0.64–2.66)	0.372	1.43 (0.65–3.15)	0.057	0.52 (0.26–1.02)	1.000	1 (0.49–2.03)
Oral antidiabetics	0.193	0.46 (0.15–1.48)	0.007	0.2 (0.06–0.65)	0.397	0.7 (0.31–1.59)	0.32	0.6 (0.23–1.62)
Insulin	0.253	0.55 (0.19–1.54)	0.349	2.1 (0.46–9.20)	0.075	4 (0.87–18.47)	0.209	2.4 (0.61–9.41)
Statins	0.038	0.25 (0.07–0.93)	0.550	0.74 (0.28–1.96)	0.008	0.13 (0.03–0.59)	0.944	0.96 (0.34–2.77)

### Follow-up at 3 months

Excellent outcome differed significantly overall and between sexes (*p*-all/W < 0.001; *p*-M = 0.013). Swiss (51.9%) vs. Polish/Ukrainian (36.5%/35.5%) men had higher rates; and overall higher than Swiss (44.1%) vs. Polish/Ukrainian (25.9%/17.6%) women. Favourable outcome differed significantly overall (*p*-all = 0.045), but not between sexes (*p*-W = 0.058; *p*-M = 0.188). Mortality did not differ significantly.

Recurrence of stroke/transient ischaemic attack (TIA) was similar. Re-hospitalization rates at 3 months showed significant differences (*p*-all < 0.001/*p*-W = 0.045/*p*-M = 0.014), with the highest re-hospitalization rate in Swiss women (18.7%) and lowest among Ukrainian men (3.4%). Use of antihypertensives, oral diabetics, and insulin was similar. Use of OACs differed significantly (*p*-all = 0.006/*p*-W = 0.001/*p*-M = 0.308) and was higher in Swiss (W: 43.8%/M: 35.8%) and Polish (W: 58.1%/M: 32.0%) and similar in Ukrainian women (W: 25.0%/M: 25.8%). Use of antiplatelets differed significantly among women (*p*-W = 0.007), but not overall and among men (*p*-M = 0.992). The highest use of antiplatelets was among Ukrainian women (69.4%) and the lowest among Polish women (39.5%). Use of statins showed significant differences overall (*p*-all < 0.001) and among both women (*p*-W < 0.001) and men (*p*-M = 0.002). The highest use of statins was among Polish women (88.4%) and the lowest among Ukrainian women (63.9%). Active smoking rates showed significant differences overall (*p* < 0.001) and among both women (*p*-W = 0.003) and men (*p*-M = 0.004). The highest rate was observed in Swiss women/men (18.8%/18.7%), while the lowest rate was among Ukrainian women/men (1.4%/3.4%).

**Fig. 1:**
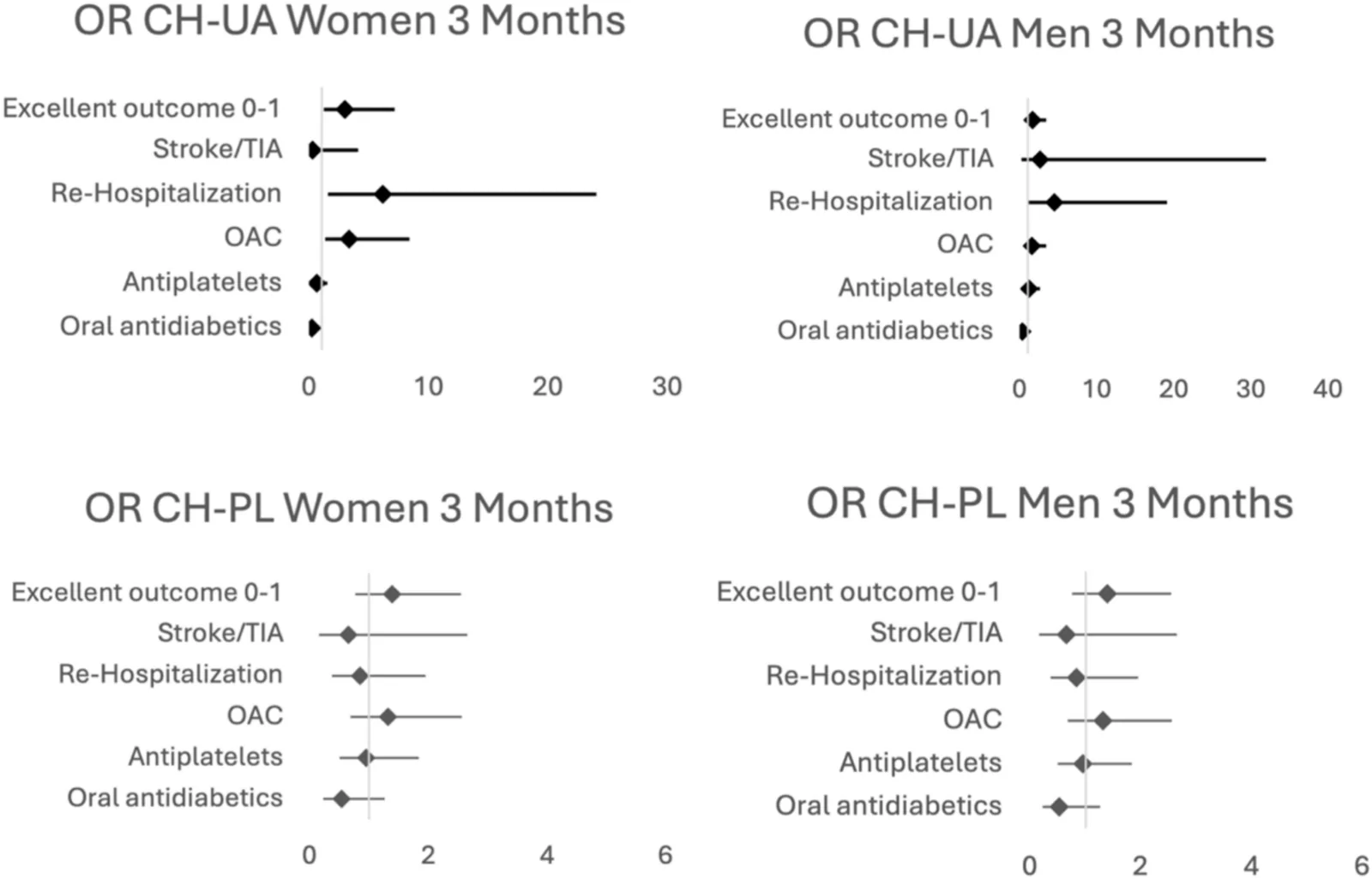
Multivariable analysis of outcome and medication at 3 months. Forrest plots of the adjusted OR CH-PL women and men at 3 months and adjusted OR CH-UA women and men at 3 months for excellent outcome, stroke/TIA, re-hospitalization, OACs, antiplatelets, and oral antidiabetics

After adjustment: Excellent outcome was significantly more likely in Swiss vs. Ukrainian women (adjusted-OR-W = 2.96/adjusted-*p*-W = 0.015). Recurrent stroke/TIA was significantly less often seen in Swiss vs. Polish women (adjusted-OR-W = 0.10/adjusted-*p*-W = 0.016). Re-hospitalization was significantly more frequent in Swiss vs. Ukrainian women (adjusted-OR-W = 6.13/adjusted-*p*-W = 0.009) and men (adjusted-OR-M = 4.45/adjusted-*p*-W = 0.044). Use of antihypertensives, insulin, and statins was similar. Use of OACs was significantly more often found in Swiss vs. Ukrainian women (adjusted-OR-W = 3.32/adjusted-*p*-W = 0.011). Use of antiplatelets was significantly more prevalent in Swiss vs. Polish women (adjusted-OR-W = 2.50/adjusted-*p*-W = 0.024). Use of oral antidiabetics was significantly less often seen in Swiss vs. Ukrainian women (adjusted-OR-W = 0.22/adjusted-*p*-W = 0.041) and men (adjusted-OR-M = 0.27/adjusted-*p*-W = 0.027). Active smoking was significantly more observed in Swiss vs. Ukrainian women adjusted-OR-W = 9.76/adjusted-*p*-W = 0.050) and men (adjusted-OR-W = 9.75/adjusted-*p*-W = 0.004). Further details are provided in Tables 4 and 5 and Fig. 1.

**Table 4:** Follow-up at 3 months according to sex

Follow-up 3 months [*n* (%)]	Women			Men			*p*-value (Pearson)
	CH	POL	UA	CH	POL	UA	
mRS							
Excellent 0–1	51 (44.1%)	14 (25.9%)	15 (17.6%)	81 (51.9%)	31 (36.5%)	33 (35.5%)	All < 0.001/women < 0.001/men = 0.013
Favourable 0–2	68 (57.6%)	21 (38.9%)	40 (47.1%)	105 (67.3%)	49 (57.6%)	65 (69.9%)	All = 0.045/women = 0.058/men = 0.188
Dead 6	21 (17.6%)	11 (20.4%)	10 (11.6%)	27 (17.3%)	11 (12.9%)	11 (10.8%)	All = 0.173/women = 0.333/men = 0.315
Stroke/TIA	2 (2.1%)	5 (10.9%)	5 (6.9%)	5 (3.9%)	4 (5.3%)	1 (1.1%)	All = 0.152/women = 0.086/men = 0.311
Re-hospitalization	17 (18.7%)	7 (15.9%)	4 (5.6%)	18 (14.6%)	12 (16.2%)	3 (3.4%)	All < 0.001/women = 0.045/men = 0.014
Medication							
Antihypertensives	70 (79.5%)	36 (83.7%)	63 (87.5%)	107 (87.0%)	66 (88.0%)	81 (91.0%)	All = 0.303/women = 0.405/men = 0.655
OACs	39 (43.8%)	25 (58.1%)	18 (25.0%)	44 (35.8%)	24 (32.0%)	23 (25.8%)	All = 0.006/women = 0.001/men = 0.308
Antiplatelets	53 (60.2%)	17 (39.5%)	50 (69.4%)	81 (65.9%)	49 (65.3%)	59 (66.3%)	All = 0.130/women = 0.007/men = 0.992
Oral antidiabetics	8 (9.0%)	6 (14.0%)	11 (15.3%)	15 (12.2%)	13 (17.3%)	15 (16.9%)	All = 0.245/women = 0.447/men = 0.517
Insulin	4 (4.5%)	5 (11.6%)	2 (2.8%)	7 (5.7%)	2 (2.7%)	2 (2.2%)	All = 0.310/women = 0.114/men = 0.361
Statins	78 (87.6%)	38 (88.4%)	46 (63.9%)	107 (87.0%)	65 (86.7%)	62 (69.7%)	All < 0.001/women < 0.001/men = 0.002
Active smoking	12 (18.8%)	4 (9.3%)	1 (1.4%)	17 (18.7%)	12 (16.2%)	3 (3.4%)	all < 0.001/women = 0.003/men = 0.004

For 47/22/9 patients from Switzerland/Poland/Ukraine, who had died until the follow-up, no data besides mRS was assessed

mRS-3 months CH: *n* = 275, data of 18 patients missing, stroke/TIA CH: *n* = 225, data of 21 patients missing, PL: *n* = 108, data of 10 patients missing, UA: *n* = 161, data of 19 patients missing, re-hospitalization CH: *n* = 214, data of 32 patients missing, UA: *n* = 161, data of 19 patients missing, medication UA: *n* = 161, data of 18 patients missing, antihypertensives CH: *n* = 211, data of 35 patients missing, OAC CH: *n* = 212, data of 34 patients missing, antiplatelets CH: *n* = 211, data of 35 patients missing, statins/oral antidiabetics CH: *n* = 212, data of 34 patients missing, insulin CH: *n* = 211, data of 35 patients missing, active smoking CH: *n* = 155, data of 91 patients missing, PL: *n* = 117, data of 1 patient missing, UA: *n* = 161, data of 19 patients missing

**Table 5:** Follow-up at 3 months according to sex

Follow-up 3 months [*n* (%)]	Women				Men			
	*p*-value (adjusted CH-PL)	Adjusted odds ratio, CH-PL 95% CI	*p*-value (adjusted CH-UA)	Adjusted odds ratio, CH-UA 95% CI	*p*-value (adjusted CH-PL)	Adjusted odds ratio, CH-PL 95% CI	*p*-value (adjusted CH-UA)	Adjusted odds ratio, CH-UA 95% CI
mRS								
Excellent 0–1	0.416	1.4 (0.62–3.15)	0.015	2.96 (1.23–7.12)	0.287	1.39 (0.76–2.55)	0.172	1.65 (0.80–3.37)
Favourable 0–2	0.401	1.39 (0.65–2.99)	0.447	1.41 (0.58–3.43)	0.958	1.02 (0.54–1.91)	0.422	0.7 (0.30–1.66)
Dead 6	0.498	1.37 (0.55–3.39)	0.972	1.03 (0.24–4.36)	0.125	1.88 (0.84–4.20)	0.649	1.33 (0.39–4.48)
Stroke/TIA	0.016	0.1 (0.02–0.66)	0.352	0.28 (0.02–4.08)	0.545	0.65 (0.16–2.65)	0.451	2.62 (0.21–31.92)
Re-hospitalization	0.945	1.04 (0.37–2.94)	0.009	6.13 (1.56–24.00)	0.691	0.84 (0.37–1.95)	0.044	4.45 (1.04–19.07)
Medication								
Antihypertensives	0.866	0.92 (0.32–2.59)	0.574	0.72 (0.23–2.28)	0.923	0.96 (0.38–2.41)	0.280	0.53 (0.17–1.68)
OAC	0.107	0.53 (0.24–1.15)	0.011	3.32 (1.31–8.38)	0.417	1.32 (0.68–2.56)	0.299	1.53 (0.69–3.42)
Antiplatelets	0.024	2.5 (1.13–5.53)	0.284	0.61 (0.25–1.51)	0.879	0.95 (0.50–1.83)	0.686	1.18 (0.54–2.57)
Oral antidiabetics	0.250	0.49 (0.14–1.66)	0.041	0.22 (0.05–0.94)	0.149	0.53 (0.22–1.26)	0.027	0.27 (0.09–0.86)
Insulin	0.227	0.42 (0.10–1.73)	0.414	2.62 (0.26–26.40)	0.392	2.1 (0.38–11.54)	0.798	0.75 (0.08–7.13)
Statins	0.802	1.17 (0.35–3.93)	0.156	0.21 (0.75–6.00)	0.869	1.08 (0.44–2.66)	0.064	2.51 (0.95–6.63)
Active smoking	0.400	1.76 (0.48–6.45)	0.05	9.76 (1.00–95.31)	0.434	1.41 (0.59–3.36)	0.004	9.75 (2.06–46.12)

## Discussion

### Summary of key findings

This study emphasizes that sex differences in stroke outcomes are influenced by a combination of biological and socioeconomic factors, associated with distinct baseline characteristics and varying access to care. The main finding was that Swiss versus Ukrainian women were three times more likely to achieve excellent outcomes.

### Baseline characteristics

Our results revealed significant differences in baseline characteristics between sexes, influenced by socioeconomic factors.

Women in Ukraine had a higher BMI than those in Poland and Switzerland, whereas men's BMI was similar. This suggests that lower socioeconomic status contributes to unhealthy weight profiles in women, potentially due to food insecurity, limited physical activity opportunities, stress, lower education and health literacy, cultural norms, and healthcare barriers [17, 24, 30, 31].

AF was highly prevalent, particularly in Polish and Ukrainian women, and likely contributed to the higher rates of cardioembolism observed in the TOAST classification in those patient groups. Given its strong association with stroke risk, targeted screening and improved management are essential, particularly in regions with limited healthcare resources [3, 16].

Lower smoking rates among women, especially in Ukraine, likely reflect cultural norms and historical trends, though underreporting may obscure its true impact on stroke risk. Therefore, culturally sensitive education and cessation programmes remain crucial.

Differences in arterial hypertension, hyperlipidaemia, previous myocardial infarction prevalence, and large artery atherosclerosis in TOAST aetiology may stem from disparities in healthcare access and genetic predisposition. Notably, the absence of small vessel occlusion and other determined stroke aetiologies and low number of cryptogenic strokes in Ukraine may partly reflect selection bias in this cohort. Beyond these regional and methodological factors, higher pre-stroke disability in women indicates a greater comorbidity burden at stroke onset.

These findings are associated with women’s increased vulnerability and the need for tailored pre-stroke care, region-specific risk reduction strategies and further research to better understand these disparities [15, 16, 24, 32].

### Symptom presentation and diagnostic delays

Our results show that Polish women had the highest NIHSS scores on admission, suggesting more severe strokes, likely due to regional healthcare differences and a higher prevalence of AF. Given AF’s strong association with cardioembolic stroke, targeted screening and management are essential [2, 3]. Analysis of LVO occlusion sites showed that their distribution between countries was neither significantly different in women (*p*-W = 0.459) nor men (*p*-M = 0.134) alone, despite a significant overall difference (*p*-all = 0.026). Consequently, LVO occlusion sites cannot fully explain the higher stroke severity observed in Polish women.

Although we did not collect direct data on stroke symptoms and time delays, they are highly plausible contributing factors. Women often present with atypical symptoms, which can complicate diagnosis and lead to treatment delays up to three times longer than in men. These pre-hospital delays directly contribute to neurological deterioration and could, therefore, significantly account for higher initial stroke severity seen in Polish women, despite their comparable LVO profile [5, 6, 33].

These findings highlight the need for targeted interventions to optimize AF management in women and, crucially, to raise awareness of sex-specific symptoms among healthcare providers and the public to reduce time-to-treatment delays.

### Imaging and treatment disparities

Contrary to earlier reports of lower MRI use in women, our data suggest a potential shift in diagnostic practices [34]. However, men in Switzerland and Poland were more frequently documented to undergo combined CT and MRI, raising concerns about potential biases in imaging protocols that may affect the quality of care for women.

Historically, women have been less likely to receive IVT and EVT, despite equivalent eligibility and demonstrated benefits for both sexes [2, 9–11, 35]. Recent evidence, however, indicates minimal or no sex-based differences in treatment rates [9, 34]. Consistent with this, our study found comparable IVT and EVT rates in men and women in Switzerland and Ukraine. Notably, in Poland, women presented with the most severe strokes and received IVT twice as often as men. Further research is needed to identify and address the factors driving these disparities in imaging and treatment practices.

### Outcome at discharge

Women tended to have worse functional outcomes than men, though country differences were more pronounced. After adjusting for stroke severity (NIHSS score) and treatment modality, the disparity between Swiss and Ukrainian patients persisted, with Swiss women and men showing markedly better excellent outcomes (adjusted-OR-W = 8.32; adjusted-OR-M = 4.80; adjusted-*p* < 0.001). Of note, pre-stroke differences in functional independence (mRS 0–1) favoured men, although data from Ukraine were unavailable for comparison. These findings highlight the role of healthcare quality and socioeconomic factors in recovery, as well as persistent sex-related vulnerabilities despite healthcare advances [15, 16].

Contrary to previous studies, we found no significant sex-based differences in discharge mortality, suggesting that robust healthcare systems may reduce acute-phase survival differences [9, 16, 36]. However, discharge destinations reflect the impact of socioeconomic factors on recovery which are likely also influenced by sex disparities. Swiss and Polish women were more often discharged to acute care with shorter hospital stays, suggesting differences in resource allocation. In contrast, Ukrainian women had the highest home discharge rates and longest hospital stays, likely due to limited post-acute care, highlighting the need for stronger support systems in low-resource settings [16, 21, 37].

### Medication at discharge and 3 months

Differences in medication use reveal key geographical and sex-specific disparities in post-stroke care. Swiss patients, particularly men, were less likely to receive antihypertensive therapy at discharge (adjusted-OR-CH-PL-M = 0.46/adjusted-*p*-CH-PL-M = 0.024; adjusted-OR-CH-UA-M = 0.07/adjusted-*p* -CH-UA-M < 0.001), likely due to lower prevalence of arterial hypertension, though prescribing practices may also have played a role. A previously reported sex disparity in statin prescriptions was also evident, indicating the need to address biases in treatment initiation [5, 38].

By 3 months, use of statins and OACs declined in Ukraine, likely due to adherence challenges, follow-up quality, and financial barriers. In Poland, the decrease in use of antiplatelets among women suggests changes in clinical decisions or therapy access issues.

Swiss women were five times less likely to receive oral antidiabetics than Ukrainian women (adjusted-OR-W = 0.22/adjusted-*p*-W = 0.041), despite similar diabetes prevalence, possibly associated with regional differences in treatment approaches, guidelines or resource availability. These findings underscore the need to address regional and sex-specific disparities in post-stroke care.

### Outcome at 3 months

Women showed worse functional outcomes at 3 months than men, with pronounced country-specific differences. Swiss compared to Ukrainian women were three times more likely to show excellent outcome, in adjusted analysis (adjusted-OR-W = 2.96/adjusted-*p*-W = 0.015). These findings suggest that women may be more vulnerable to socioeconomic disadvantages than men [5, 15, 16, 21, 38]. Interestingly, in a prior analysis without sex dichotomization, the 3-month excellent outcome difference between Switzerland and Ukraine was only twofold [26]. Mortality at 3 months, however, did not differ significantly between sexes or countries.

At 3 months, recurrent stroke/TIA was ten times less frequent in Swiss versus Polish women (adjusted-OR-W = 0.10/adjusted-*p*-W = 0.016), likely due to higher baseline vascular risk and greater pre-/post-stroke morbidity (mRS) in Polish women. Previous studies have linked recurrent stroke to lower socioeconomic status, highlighting that even in high-income countries, socioeconomic disparities disproportionately affect women [39, 40].

### Strengths

The study's key strength is its multicentre, prospective design, allowing direct hospital comparisons across three distinct socioeconomic contexts. It provides novel, direct evidence of how sex and socioeconomic factors jointly influence stroke risk, care, and outcomes in diverse European cohorts. By integrating clinical and socioeconomic data, the study fills an important gap in cross-country stroke research and supports the European Stroke Action Plan’s call for tailored, equitable care [24].

### Limitations

The main limitations of this study include patient consent issues, loss to follow-up, and missing data, especially on secondary prevention (e.g., smoking status, medication adherence). Further only information on biological sex was collected; no data on self-identified gender or gender roles was available. Generalizability is restricted, as participating centres exceed national quality standards, potentially underestimating disparities in less affluent countries. Individual socioeconomic factors such as income, education status, or employment were not assessed. Moreover, 2019 data may not reflect current conditions due to treatment advances, COVID-19, and the war in Ukraine. Future studies should use recent data to track evolving stroke care trends.

## Conclusion

This study highlights sex differences in stroke risk, treatment, and outcomes across Switzerland, Poland, and Ukraine, associated with diverse socioeconomic contexts. Swiss women were three times more likely to achieve excellent outcomes at 3 months compared to Ukrainian women, emphasizing the impact of prevention strategies, healthcare infrastructure, and resources on recovery. These findings underscore the need for sex-, gender-, and socioeconomic-specific approaches to stroke prevention and management, with tailored interventions to address disparities, particularly in women and regions with lower socioeconomic conditions. The European Stroke Action Plan 2018–2030 represents a crucial step towards more equitable stroke care, including attention to sex-specific healthcare needs.

## Data Availability

Raw data of all patients included in this study can be made available upon request to the corresponding author and after clearance by the local ethics committee.
